# TRPA1 as a promising target in ischemia/reperfusion: A comprehensive review

**DOI:** 10.22038/IJBMS.2023.74590.16198

**Published:** 2024

**Authors:** Azin Alizadehasl, Maryam Sadat Alavi, Mohaddeseh Sadat Alavi, Ali Roohbakhsh

**Affiliations:** 1 Cardio-Oncology Research Center, Rajaie Cardiovascular Medical and Research Center, Iran University of Medical Sciences, Tehran, Iran; 2 Department of Echocardiography, Rajaie Cardiovascular Medical and Research Center, Iran University of Medical Sciences, Tehran, Iran; 3 Pharmacological Research Center of Medicinal Plants, Mashhad University of Medical Sciences, Mashhad, Iran; 4 Pharmaceutical Research Center, Pharmaceutical Technology Institute, Mashhad University of Medical Sciences, Mashhad, Iran; 5 Department of Pharmacodynamics and Toxicology, School of Pharmacy, Mashhad University of Medical Sciences, Mashhad, Iran

**Keywords:** Inflammation, Ischemia, Reactive oxygen species, Transient receptor potential – channels, TRPA1 cation channel

## Abstract

Ischemic disorders, including myocardial infarction, cerebral ischemia, and peripheral vascular impairment, are the main common reasons for debilitating diseases and death in Western cultures. Ischemia occurs when blood circulation is reduced in tissues. Reperfusion, although commanded to return oxygen to ischemic tissues, generates paradoxical tissue responses. The responses include generating reactive oxygen species (ROS), stimulating inflammatory responses in ischemic organs, endoplasmic reticulum stress, and the expansion of postischemic capillary no-reflow, which intensifies organ damage. Multiple pathologic processes contribute to ischemia/reperfusion; therefore, targeting different pathologic processes may yield an effective therapeutic approach. Transient Receptor Potential A1 (TRPA1) belongs to the TRP family of ion channels, detects a broad range of chemicals, and promotes the transduction of noxious stimuli, e.g., methylglyoxal, ROS, and acrolein effects are attributed to the channel’s sensitivity to intracellular calcium elevation or phosphoinositol phosphate modulation. Hypoxia and ischemia are associated with oxidative stress, which activates the TRPA1 channel. This review describes the role of TRPA1 and its related mechanisms that contribute to ischemia/reperfusion. Relevant articles were searched from PubMed, Scopus, Web of Sciences, and Google Scholar electronic databases, up to the end of August 2023. Based on the evidence presented here, TRPA1 may have protective or deteriorative functions during the ischemia/reperfusion process. Its function depends on the activation level, the ischemic region, the extent of lesions, and the duration of ischemia.

## Introduction

Ischemia occurs whenever the blood supply to tissues is interrupted, which means a reduction in oxygen level in that area. Reoxygenation of the organs is called reperfusion (1, 2). Ischemia mainly affects oxygen-dependent organs such as the kidney, liver, heart, and brain (3). Therefore, ischemia/reperfusion (I/R) contributes to the pathology of a wide range of diseases such as renal ischemia, myocardial infarction, peripheral vascular impairment, cerebral ischemia, and stroke (4). Multiple factors can cause ischemia including embolism, especially cardioembolism, large vessel disease, or occlusion of small vessels in the brain (5).

The underlying processes responsible for I/R-induced cellular damage include a rise in Ca^2+ ^levels, impairment of energy balance, and generation of free radicals which bind to cellular macromolecules and promote oxidative damage (6). Other factors contributing to cell injuries caused by I/R include activation of reactive oxygen species (ROS)-dependent pathways such as inflammation and apoptosis (7).

The transient receptor potential ankyrin 1 (TRPA1), is a nonselective cation channel that is permeable to Ca^2+^ that enhances intracellular Ca^2+ ^levels (8). TRPA1 is located in the primary afferent neurons of the trigeminal and dorsal root ganglion (DRG) and is involved in pain-related behaviors (9). In addition, a growing body of evidence indicates that TRPA1 channels exist in non-neuronal organs including the heart, kidney, liver, lungs, and intestine (10). TRPA1 is stimulated by a wide range of environmental irritants containing natural compounds allyl isothiocyanate (AITC), wintergreen oil, mustard oil, cinnamaldehyde, allicin, and cannabidiol and chemical compounds, such as ASP-7663, optovin, acrolein, propofol, and lidocaine (11, 12). It also plays a fundamental role in sensing noxious cold induced by internal stimuli such as oxidative stress products and inflammatory cytokines (13). Moreover, HC-030031, AP-18, and A-967079 are TRPA1 antagonists that selectively bind to this channel (Table 1) (14). 

There is a link between hypoxia and excessive ROS production and overproduction of unsaturated aldehydes, including 4-hydroxy-2-nonenal (4-HNE), an endogenous activator of the TRPA1 channel (15). In other words, TRPA1 is activated by free radicals during ischemia through oxidation or cysteine residue modification on its N-terminal (10, 16). During I/R, ROS production promotes tissue inflammation and activates immune responses (17). The environmental cells in the area of inflammation release inflammatory cytokines and chemokines, like leukotrienes, tumor necrosis factor α (TNF-α), interleukins (IL), prostaglandins, and proteases (18). Macrophages also express TRPA1 and it has a regulatory function along with other inflammatory mediators (19). However, TRPA1’s role in stroke is controversial. Despite some researchers supporting TRPA1 as a promising target for I/R injury (20, 21), other studies suggest that TRPA1 activation promotes ischemic injury (16, 22). This review discusses TRPA1 channel involvement in I/R damage in different organs and its detailed mechanisms.

***Methods***

A bibliographic search was done using the following databases: PubMed, Scopus, Web of Science, and Google Scholar to identify studies and papers published in English using the following keywords: ‘ischemic stroke’, ‘stroke’, ‘myocardial infarction’, ‘cerebral ischemia’, ‘heart ischemia’, ‘peripheral vascular disease’, ‘peripheral ischemia’, ‘renal ischemia’, ‘retinal ischemia’, ‘lung ischemia’ in combination with ‘TRPA1’. Articles were judged for inclusion based on the relevance of the title and abstract and excluded if they did not fit the topic. No time limit (up to August 2023) was considered in the current review.

***TRPA1 and cerebral ischemia***

Disruption of regional blood circulation in the brain can rapidly induce irreversible neuronal damage. The unique capabilities of cerebral blood vessels and circulation allow them to adjust to varying physiological and pathological conditions to maintain optimal perfusion and decrease brain injury (23). Global cerebral I/R activates inflammation that extends neuropathic pain. By affecting the spinothalamic pathways, cerebral I/R causes complex maladaptive sensory neuron changes (24). TRPA1 signaling deficiency in the endothelium of the cerebral arteries intensified cerebral infarctions in the mice model of permanent middle cerebral artery occlusion (MCAO). Further, pharmacological augmentation of TRPA1 reduced infarctions, an effect diminished by the loss of endothelial TRPA1 channels (25). Isoflurane, the anesthetic medicine used during MCAO surgeries potentiates TRPA1 activity. Pires and co-workers suggested that isoflurane, by activation of TRPA1, augmented cerebral arterial dilation and decreased ischemic damage (25).

In contrast, other studies showed that pharmacological activation of TRPA1 increased brain tissue loss during an ischemic stroke. Zaki *et al*. showed that cilostazol alleviated neuropathic pain after global cerebral I/Rcaused by bilateral carotid occlusion in rats (26). They demonstrated that cilostazol treatment deactivated the nucleotide-binding domain-like receptor protein 3 (NLRP3) inflammasome, activated the nuclear factor erythroid 2–related factor 2 (Nrf2) axis, and promoted neuronal survival and dopamine neurotransmission (26). Furthermore, cerebral I/R increased the cortical content of TRPA1. Cilostazol significantly inhibited TRPA1 and excitotoxicity (26). Researchers believe that TRPA1 inhibition through the activation of the neuronal protein kinase B (Akt) survival cascade concurrent with brain-derived neurotrophic factor (BDNF) and enhancing the Nrf2 axis are the mechanisms of neuroprotective effects of cilostazol (26).

Recent evidence indicates that during simulated ischemia, TRPA1 channels induce myelin damage and white matter loss (27). Also, carvacrol and JT010 via TRPA1 activation suppressed myelination and promoted myelin loss in cortical slices (28). A-967079, a selective TRPA1 antagonist, decreased basal oligodendrocyte Ca^2+^ levels and increased action potential in the optic nerve (29). Conversely, TRPA1 agonists (polygodial and AITC) reduced the optic nerve action potential amplitude that was inhibited by A-967079. Researchers found that TRPA1 inhibition prevented action potential loss during OGD and improved recovery (29). In other words, they indicated that glial TRPA1 controls neuronal excitability in the brain in both pathological and physiological situations (29). Zhou *et al*. found that TRPA1 has a key role during simulated ischemia-induced demyelination. Also, they showed that post-treatment with desflurane attenuated hypoxic-ischemic brain injury by reducing TRPA1 in rat pups (30). TRPA1 inhibition with HC-030031 reduced perinatal hypoxic-ischemic brain tissue damage and reversed learning and memory impairment (30). An overview of the role of TRPA1 in different I/R experimental models is presented in Table 2. 

***TRPA1 and cardiac ischemia***

Myocardial infarction is a major reason for mortality and morbidity worldwide. For the treatment of infarction, early reperfusion of the blocked coronary artery is essential to reduce myocardial injury. But, reperfusion may lead to extra damage to the myocardium (31).

Lu and colleagues indicated that TRPA1 agonists, ASP-7663, and optovin, reduced cardiomyocyte cell death when given during reperfusion in a rat model of cardiac I/R injury (20). However, unlike the other TRPA1 activators, cinnamaldehyde did not impact myocardial infarct size. The researchers suggested that the opioid-caused decrease in myocardial infarct size was mediated by TRPA1 as its pharmacological inhibition prevented morphine’s ability to reduce infarction size (20). They also suggested that TRPA1 activation by ASP-7663 and optovin before or during hypoxia-reoxygenation protected adult rat primary cardiomyocytes from injury. This effect was blocked by AP-18 and TCS-5861528 (20). It is worth mentioning that TRPA1 has a functional interaction with the opioid system, as it can affect morphine-induced reward and analgesia (32).

Topical painkiller creams containing natural products may enter the bloodstream circulation after application and show systemic effects. Wu and co-workers supposed that topical application of IcyHot cream containing methyl salicylate affected myocardial infarct size (10). By increasing methyl salicylate levels in the blood, IceHot topical cream protected rats from cardiac I/R that was mediated by TRPA1 (10). Pretreatment with TRPA1 inhibitors (TCS-5861528 and AP-18) blocked IcyHot-induced infarct size reduction (10). Also, isolated adult cardiomyocytes exposed to methyl salicylate during reoxygenation exhibited lower cell death. Treatment or pre-treatment with the TRPA1 antagonists, AP-18 or TCS-5861528, suppressed cardioprotection by methyl salicylate (10). 

**Figure 1:**
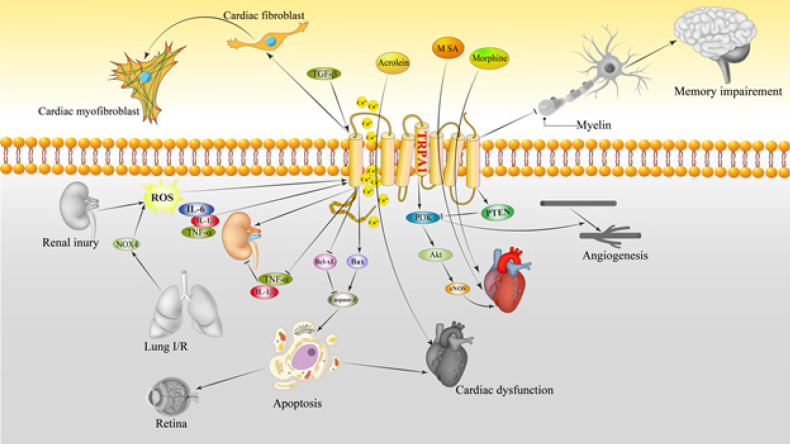
A schematic illustration of TRPA1-mediated effects during I/R on various organs. TRPA1 may have both beneficial and detrimental effects on I/R. Black and white illustrations of organs show detrimental effects while color illustrations show beneficial effects of TRPA1. → presents the promote/activate and ⊥ presents the inhibitory/suppressive effects

TRPA1 activation with AITC promotes cardiomyocyte survival following an ischemic insult. AITC, concentration- and time-dependently, enhanced cardiomyocyte contractile function via Akt and endothelial nitric oxide synthases (eNOS) phosphorylation and subsequent NO production augmentation (33). Moreover, TRPA1 increased cardiomyocyte contractile function by enhancing Ca^2+ ^in cardiomyocytes independently of Akt and eNOS activation mechanisms (33) (Figure 1). 

Cardiac fibroblasts are an important cellular element of post-myocardial infarction left ventricular remodeling (34). In cardiac injury situations, cardiac fibroblasts undergo programmed conversion into cardiac myofibroblasts. TRPA1 overexpression significantly activated cardiac myofibroblasts transformation, while TRPA1 deficient cardiac fibroblasts were resistant to transforming growth factor-*β* (TGF-*β*)-caused transdifferentiation (21). TGF-*β* increased TRPA1 expression, which stimulated the Ca^2+^ responsive activation of calcineurin (CaN). Moreover, dual-specificity tyrosine-regulated kinase-1a (DYRK1A) modulated the CaN-mediated nuclear factor of activated T cells (NFAT) translocation and TRPA1-dependent transdifferentiation (21). 

A recently published study found that activating TRPA1 pharmacologically with JT010 or inhibiting it with A-967079 did not change infarct size in rats (31). In addition, *TRPA1 *deletion in C57BL/6 mice could not protect the heart from ischemia. However, in a co-culture of primary adult murine sensory neurons with cardiomyocytes, cardiomyocyte survival probability increased slightly when they were challenged by I/R which was attributed to TRPA1 (31). In contrast, Conklin *et al.* revealed that TRPA1 is expressed on the sarcolemma and cardiomyocytes’ intercalated disks and is a potential target for acrolein, a main product of lipid peroxidation (35). They reported that acrolein-induced infarction was substantially reduced in cardiomyocytes isolated from TRPA1-null mice than in wild-type. Acrolein-induced I/R increased Ca^2+^ overload and hypercontraction that was notably diminished by HC-030031 (35). The protective effect of HC-030031 was equivalent to that produced by SN-6, a sodium/calcium exchange inhibitor, confirming the role of Ca^2+^ accumulation in acrolein-injured cardiomyocytes (35). The researchers suggested that HC-030031 treatment significantly inhibited myocardial infarction-triggered cardiac dysfunction, diminished cell apoptosis and cardiac fibrosis, and increased angiogenesis at the infarct border in C57BL/6 mice (22). It also declined phosphatase and tensin homolog (PTEN) expressions and augmented phosphorylated Akt expression in the myocardium isolated from C57BL/6 mice and human umbilical vein endothelial cells (HUVECs) (22). HUVECs pretreated with a phosphoinositide 3-kinase (PI3K) inhibitor (LY294002), almost completely abolished HC-030031-mediated migration in HUVECs. In addition, researchers observed that HC-030031 treatment inhibited myocardial infarction increased Bax expression, and enhanced Bcl-xL expression (22). As a result, TRPA1 inhibition activated the PI3K/Akt pathway by down-regulating PTEN expression in the myocardium. TRPA1 inhibition also increased vascular endothelial growth factor (VEGF) levels in endothelial cells. In other words, PI3K suppression abolished TRPA1 inhibition-promoted angiogenesis in HUVECs (22). A study by Üstünel *et al*. assessed the effects of iloprost (anti-oxidant) and a β3 adrenergic receptor agonist on both TRPA1 and TRPC1 ion channels during cardiac I/R injury in male Wistar rats (36). Their results indicated that total oxidant, TRPA1, and TRPC1 levels were markedly enhanced in the I/R group. However, treatment with either iloprost or β3 adrenergic receptor agonists did not alter TRPA1 levels compared to the I/R group (36).

***TRPA1 and peripheral ischemia***

In humans, limb ischemia occurs as peripheral artery disease (PAD). Subsequent reperfusion after limb ischemia may cause dysesthesia associated with pain and numbness. Evidence recommends that oxidant-induced stimulation of TRPA1 during ischemic episodes is involved in pain syndromes and a variety of pathologic cutaneous sensations such as itching and peripheral post-ischemic licking (37, 38). 

Complex regional pain syndrome type 1 (CRPS1) is a disease that causes severe pain and usually occurs after surgery, fractures, as well as limb ischemia. Experimental models of semi-permanent hindlimb ischemia are commonly applied for PAD pathophysiology assessment, as well as ischemic pain (38). Chronic post-ischemia pain (CPIP) is a useful experimental model of CRPS1 induced by I/R. Klafke *et al*. showed that TRPA1 activation is appropriate for CRPS1/CPIP-caused acute and chronic pain (39). They induced both acute and chronic CPIP using the I/R hindpaw rat model. In both acute and chronic episodes of I/R, cold and mechanical allodynia were increased activation of inflammatory pathways and oxidative stress signaling occurred. Administration of HC-030031 strongly prevented mechanical and cold allodynia (39).

Similarly, mechanical and cold allodynia in the hindlimb of mice suffering from CPIP were permanently attenuated by TRPA1 ablation (40). Pharmacological inhibition of TRPA1 with A-967079 and HC-030031 antagonists or α-lipoic acid also transiently inhibited allodynia (40). The percentage of macrophages (F4/80^+^ cells) and 4-HNE oxidative stress marker in the injured tibial nerve was higher following I/R induction. In addition, the injured nerve trunks of TRPA1-null mice did not show any increase in macrophage percentage or 4-HNE (40). Outstandingly, the knockdown of Schwann cells’ TRPA1 resulted in decreased macrophage infiltration, 4-HNE, and mechanical and cold allodynia in mice. Moreover, CPIP mice were permanently protected from neuroinflammation and allodynia by TRPA1 antagonists (40). 

Dysesthesia is an abnormal sensation that usually occurs with peripheral neuropathy or vascular damage. Peripheral postischemic dysesthesia happens when an ischemic tissue is perfused. Researchers observed that TRPA1 plays a role in dysesthesia-like behaviors provoked by transient hindlimb I/R in male mice (38, 41). Sasaki *et al*. hypothesized that postischemic dysesthesia might induce licking behaviors in animals (42). They investigated their hypothesis in a mouse model of hindpaw ischemia. Then, capsaicin or hydrogen peroxide solutions were injected into the hindpaw to induce licking (42). Activation of TRPA1 channels provoked peripheral post-ischemic dysesthesia, and post-hindpaw licking, mediated by myelinated afferent fibers in male C57BL/6 mice. The researchers also found that lickings were alleviated following ROS scavengers (N-acetyl-L-cysteine) or HC-030031 pretreatment (42). Similar to the previous study, So *et al.* found that licking behavior was inhibited by ROS scavengers including 4-hydroxy-2,2,6,6-tetramethyl-1-piperidinyloxy, and α-phenyl-*tert*-butyl nitrone (38). Under TRPA1 deficiency condition or using a TRPA1 antagonist (10-50 mg/kg HC-030031) similar protective results were observed (38). Similar results were observed in a diabetic peripheral neuropathy model using streptozotocin in C57BL/6J (43). The hypersensitivity to mechanical stimuli and cold in diabetic mice peaked two weeks after streptozotocin injection. It was likely that hypersensitivity was accompanied by decreased skin blood flow in the hindpaw (43). 

Hiyama and co-workers demonstrated that streptozotocin-caused cold hypersensitivity was attenuated by HC-030031 or TRPA1 ablation, while mechanical hypersensitivity did not alter (43). Moreover, two weeks after streptozotocin administration, intraplantar injections of the TRPA1 agonist, AITC, evoked nocifensive behaviors. Tadalafil, a phosphodiesterase 5 inhibitor, suppressed nocifensive behavior and cold hypersensitivity induced by AITC and streptozotocin, respectively, and diminished blood flow to the skin was recovered (43). 

Femoral artery ligation in rats acts as a valuable preclinical model to investigate human PAD. As shown by Xing *et al*., 24-72 hr after femoral artery occlusion, TRPA1 protein level was up-regulated in DRG, especially in the afferent nerves of C-fiber (44). 

Administration of AITC increased the amplitudes of inward current responses in DRG neurons, dose-dependently. Femoral occlusion increased AITC-sensitive DRG neurons percentage which was inhibited by HC-030031 (45). TRPA1 co-localization with proteinase-activated receptor 2 (PAR2) is observed in rat DRG neurons. PAR2 function augments TRPA1-induced currents in DRG neurons. Furthermore, rats with a femoral arterial occlusion showed PAR2 expression enhancement in DRG neurons. Moreover, the PAR2 agonist SL-NH2 enhanced TRPA1 amplitude in DRG neurons of an occluded limb, while the PAR antagonist FSLLRY-NH2 attenuated TRPA1 stimulation (46). Phospholipase C (PLC) and phosphatidylinositol 4,5-bisphosphate (PIP2), the downstream signaling pathways, were involved in TRPA1 current regulation by PAR2. Mitogen-activated protein kinase (MAPK) and PI3K/Akt signaling pathways were likely involved in PAR2 activation (46).

In an *in vitro *model*, *TRPA1-expressing cells and DRG neurons from mice were exposed to hypoxia. The cells were then treated with hydrogen peroxide with reoxygenation to induce I/R (38). The finding showed that TRPA1 was activated by both hydrogen peroxide and hyperoxia, via oxidative modification at the TRPA1 channel N-terminal. Furthermore, TRPA1 deletion in DRG neurons inhibited hydrogen peroxide-evoked responses, even after pretreatment with hypoxia (38). The researchers believed that TRPA1 sensitivity was selectively increased by hypoxia in DRG sensory neurons, but not in other ROS-sensitive channels (38). Acrolein is a lipid peroxidation product with greater reactivity and neurotoxicity than malondialdehyde and is a known TRPA1 activator (47). Previous studies indicated that acrolein is associated with post-I/R spinal cord injury hypersensitivity by overactivation, up-regulation, and sensitization of TRPA1 in DRG sensory neurons. Phenelzine, a known aldehyde scavenger, significantly alleviated post-I/R spinal cord injury hypersensitivity, decreased acrolein, inhibited TPRA1 up-regulation, and promoted motor neurons’ survival (48).

***TRPA1 and retinal ischemia***

There are several vision-threatening diseases caused by retinal I/R, including retinal vein occlusion and specifically glaucoma. Neuronal depolarization, Ca^2+^ influx, oxidative stress, and inflammation all contribute to retinal ischemia (49). 

 Genetic loss or pharmacological inhibition of TRPA1 protected retinal cells from ischemia-induced damage seen in normal mice (50). In a C57BL/6J mice model of I/R retinal damage, TRPA1 ablation or administration of eye drops consisting of TRPA1 antagonists (HC-030031 and A-967079) mitigated activated caspase-3, decreased retinal cell death, and maintained retinal tissue thickness (50). Treatment with α-lipoic acid provided similar protection, indicating that the damage of tissue is entirely caused by both oxidative stress and TRPA1. However, TRPV1 or TRPV4 ablation did not induce protective effects on I/R damage (50). In accordance, Araujo *et al.* confirmed TRPA1 expression in the chick retina and found a rise in TRPA1 content after oxygen and glucose deprivation (OGD) (51). TRPA1 activation by mustard oil did not change retinal lactate dehydrogenase (LDH) release caused by OGD. However, TRPA1 inhibition by HC-030031 avoided cellular LDH extravasation in ischemic conditions (51). Also, mustard oil combined with WIN55212-2, the cannabinoid receptor agonist, dramatically increased LDH release. Interestingly, treatment with AM251 and O-2050 (cannabinoid receptor 1 antagonists) or AM630 (cannabinoid receptor 2 antagonist) prevented cell death induced by WIN55212-2/mustard oil (51). Another study indicated that TRPA1 selective antagonist, A-967079 increased action potentials recorded in optic nerves isolated from C57BL/6J mice. In the OGD model, reduction in action potential amplitude has been attributed to elevated Ca^2+^ influx. In agreement, during OGD, TRPA1 inhibition prevented action potential loss in the optic nerve (29).

***TRPA1 and renal ischemia***

**Renal I/R injury is a common cause of renal dysfunction and acute kidney injury (AKI). AKI is linked to remarkable morbidity and mortality during patients’ hospitalizations (** 52).

Following I/R injury, TRPA1-knockout mice exhibited more worsened biochemical and pathological signs of AKI compared to the intact mice (37). It is well known that M1 macrophages facilitate the inflammation process while M2 macrophages exhibit an anti-inflammatory phenotype and are responsible for tissue repair and scar formation. In a study by Ma and colleagues, *TRPA1* ablation raised TNF-α and IL-1β levels generated by M1 macrophages but not IL-10 and TGF-β produced by M2 macrophages (37). They showed that *TRPA1 *gene ablation exacerbated macrophage infiltration renal inflammation and injury in mice after I/R. Activation of TRPA1 caused protective effects against I/R-caused AKI through control of the macrophage-mediated inflammatory pathway (37). In contrast to the previous study, in another animal model of renal I/R, tubular TRPA1 expression was increased. Genetic deletion of *TRPA1* in mice led to less I/R-induced tubular dysfunction, inflammation, oxidative stress, and kidney dysfunction compared to normal animals (53). On the other hand, exposure of human kidney 2 (HK-2) cells to hypoxia-reoxygenation damage increased TRPA1 expression (53). Additionally, it was indicated that the tubular injury activated TRPA1, ROS-dependently, and elevated intracellular Ca^2+^ levels, enhanced NADPH oxidase, activated MAPK/nuclear factor-κB (NF-κB) signaling, and raised IL-8 levels. These effects were suppressed by inhibition of TRPA1 with HC-030031 or *TRPA1 *gene silencing. Consequently, renal tubular TRPA1 serves as a sensor for oxidative stress and a key modulator of IL-8 transcription (53) (Figure 1).

***TRPA1 and lung ischemia***

Lung I/R damage is a major complication that happens after cardiac bypass surgery or lung transplantation. As a result of the dual blood supply system and oxygen availability, the pathology of pulmonary I/R is more complex than any other organ (54).

Lung sensory nerves have a vital role in controlling respiratory functions to maintain homeostasis. A large number of the vagus nerve afferents respond to inflammatory cytokines, oxidative stress products, and noxious stimuli (55). In pathological conditions, TRPA1 activation in sensory nerves mediates neurogenic inflammation. In a rat model of lung I/R injury, expressions of oxidative stress products (8-isoprostaglandin F2α and 8-hydroxy-20-deoxyguanosine) were augmented in the commissural nucleus of the solitary tract (56). Moreover, the anti-oxidant transcription factor Nrf2 was down-regulated in the brain stem while NADPH oxidase 4 (NOX4) and TRPA1 were up-regulated. Blocking NOX4 decreased oxidative stress products in the commissural nucleus of the solitary tract and suppressed up-regulation of TRPA1 following lung I/R injury (56). In addition, pharmacological inhibition of proinflammatory cytokines, such as IL-1β, IL-6, and TNF-α diminished TRPA1 up-regulation in sensory nerves following lung I/R injury (56). In agreement with the previous study, it was indicated that sensory proteinase-activated receptor-2 (PAR2) and TRPA1 were up-regulated in lung I/R. PAR2 inhibition by FSLLRY-NH2 mitigated TRPA1 up-regulation via intracellular p38-MAPK and c-Jun N-terminal kinases (JNK) signaling pathways. Moreover, blocking individual proinflammatory cytokines receptors decreased PAR2 and TRPA1 in the pulmonary vagal afferent nerves (57). 

**Table 1:** List of TRPA1 agonists and antagonists mentioned in the present article

**Product name**	**Action on TRPA1 channel**	**IUPAC name**
Acrolein	**Agonist**	prop-2-enal
Allyl isothiocyanate (AITC)		3-isothiocyanatoprop-1-ene
ASP-7663		(2*E*)-2-[7-Fluoro-1,2-dihydro-1-(2-methylpropyl)-2-oxo-3*H*-indol-3-ylidene]acetic acid
Cinnamaldehyde		(E)-3-phenylprop-2-enal
JT010		2-Chloro-*N*-[4-(4-methoxyphenyl)-2-thiazolyl]-*N*-(3-methoxypropyl)acetamide
Optovin		5-[[2,5-Dimethyl-1-(3-pyridinyl)-1*H*-pyrrol-3-yl]methylene]-2-thioxo-4-thiazolidinone
A-967079	**Antagonist**	(NZ)-N-[(E)-1-(4-fluorophenyl)-2-methylpent-1-en-3-ylidene]hydroxylamine
AP-18		(NZ)-N-[(E)-4-(4-chlorophenyl)-3-methylbut-3-en-2-ylidene]hydroxylamine
HC-030031		2-(1,3-dimethyl-2,6-dioxo-1,2,3,6-tetrahydro-7H-purin-7-yl)-N-(4-isopropylphenyl)acetamide

TRPA1: Transient receptor potential A1

**Table 2:** An overview of the role of TRPA1 in different ischemic/reperfusion (I/R) experimental models

*Tissue*	*Model*	*Study design*	*Effect*	*Reference*
***Brain***	Oligodendrocytes isolated from Sprague-Dawley rats TRPA1-knockout HC-030031, 80 μM, A-9670791, 10 μM Reduced myelin damage in ischemia			(27)
	I/R spinal cord injury in male Sprague-Dawley rats Acrolein Induced hyperalgesia via activation and upregulation of TRPA1			(48)
	Cerebral arteries isolated from TRPA1-knockout mice MCAO model in mice 4-hydroxynonenal (1 µM) and hypoxia A967079, 1 µM Cinnamaldehyde, 50 mg/kg Increased TRPA1 activity in the cerebral endothelium Increased infarct size Reduced infarct size			(25)
	Organotypic cortical slice cultures from C57BL/6J mice JT010 (10 nM), Carvacrol (50 μM), A-967079, 20 Μm Induced myelin damage prevented demyelination promoted remyelination			(28)
	OGD in optic nerve isolated from C57BL/6J mice HC-030031 (100 μM), A-967079 (20 μM) Improved the recovery			(29)
	Bilateral carotid occlusion in rats Cilostazol, 50 mg/kg Inhibited TRPA1 and excitotoxicity			(26)
	Perinatal hypoxic-ischemic brain injury in Sprague-Dawley rats HC-030031 or desflurane Reduced brain tissue loss and impairment of learning and memory			(30)
***Peripheral tissues***	Hindlimb I/R in mice HC-030031, 30-50 mg/kg or TRPA1-knockout Inhibited painful dysesthesia			(38)
	Hind-paw I/R in C57BL/6 mice HC-030031, 30-100 mg/kg Inhibited postischemic licking			(42)
	Hindpaw I/R in rats HC-030031 Reduced mechanical and cold allodynia			(39)
	Hindlimb I/R in mice TRPA1-knockout, HC-030031(300 mg/kg), A-967079 (100 mg/kg) Reduced mechanical and cold allodynia			(40)
	Femoral artery occlusion AITC (10-40 μg/kg)	HC-030031(10 µM) I/R up-regulated TRPA1 AITC amplified sympathetic responsiveness after injury	Inhibited AITC-induced currents	(44)
	Femoral artery occlusion SL-NH2 (100 µM, PAR2 agonist)	FSLLRY-NH2 (20 µg/kg, PAR antagonist) Increased the amplitude of TRPA1 currents	attenuated TRPA1 stimulation	(46)
***Eye***	Chick retinal ischemia induced by OGD WIN55212-2, AM251, O-2050, and AM630 (all 10 µM) Prevented the extravasation of cellular LDH			(51)
	Retinal ischemia in C57BL/6J mice TRPA1-knockout HC-030031, A-967079, 5 µM Protected from the ischemic damage			(50)
***Kidney***	Renal tubular I/R in mice TRPA1-knockout Decreased renal tubular injury			(53)
***Lung***	Pulmonary vagal afferent nerves isolated from lung I/R of rats --- Up-regulation of TRPA1			(57)

AITC: Allyl isothiocyanate; Akt: Protein kinase B; eNOS: endothelial nitric oxide synthases; HUVECs: Human umbilical vein endothelial cells; IP: Interaperitoneal; IV: Intravenous; I/R: Ischemic/reperfusion; LAD: Left anterior descending coronary artery; LDH: Lactate dehydrogenase; OGD: Oxygen and glucose deprivation; PI3K: Phosphoinositide 3-kinase; MCAO: Middle cerebral artery occlusion; TRPA1: Transient receptor potential ankyrin 1

Akt: Ak strain transforming or protein kinase B; Bcl-xl: B-cell lymphoma-extra large; eNOS: endothelial nitric oxide synthase; I/R: Ischemia/reperfusion; IL-1β: Interleukin-1 beta; M SA: Methyl salicylate; NOX2: NADPH oxidase 2; PI3K: Phosphoinositide 3-kinases, PTEN: Phosphatase and tensin homolog; ROS: Reactive oxygen species; TGF-β: Transforming growth factor-β; TNF-α: Tumor necrosis factor-α

## Discussion

Ischemia in the heart, brain, or other major organs often has detrimental results and may be life-threatening. There is link between I/R and the pathology of renal ischemia, lung ischemia, myocardial infarction, peripheral vascular impairment, cerebral and retinal ischemia. As TRP channels have permeability to Ca^2+^ and other cations, it is expected that they have a role in cytotoxicity and organ damage following I/R. Similar to many other TRP channels, TRPA1 has been implicated in I/R in various organs. However, contradictory results exist. The controversies may arise as a result of different methods, animals, or organs.

In hindlimb ischemia and peripheral neuropathy, as experimental models of PAD, spontaneous pain-associated behavior during reperfusion was decreased following TRPA1 genetic ablation and pharmacological inhibition (39, 41, 43). In cardiac ischemia, most of the damage happens during reperfusion injury, when an excessive amount of oxidative stress destroys cardiac tissue. Overload of Ca^2+^ admitted via TRPA1, may aggravate tissue injury. In the Conklin *et al.* study, TRPA1-null mice had smaller infarctions. TRPA1 inhibition also reduced cardiomyocyte damage (35). Animal studies suggest that TRPA1 may exacerbate lung ischemic injury by activating inflammatory mediators and oxidative stress pathways (56). In contrast, some *in vitro* studies showed that TRPA1 activation promoted cardiomyocyte survival following ischemic insults (10, 33). TRPA1 agonist also enhanced cardiomyocyte contractile function via Akt phosphorylation and enhanced Ca^2+ ^in cardiomyocytes (33). 

In the brain, ischemia causes acidosis, TRPA1 activation, and intracellular Ca^2+^ load (27). TRPA1-knockout animals showed low levels of ischemic damage that were inhibited by various TRPA1 antagonists. However, similar to previously mentioned controversies, the neuroprotective influence of TRPA1 in cerebral ischemia should be considered. As an example, in an experimental model of cerebral ischemia, endothelial cell-specific TRPA1 null rodents exhibited larger infarcts, indicating that endogenous agonist activation of TRPA1 led to protective effects. TRPA null rodents displayed higher infarct sizes, supporting the view that TRPA1 activation by endogenous agonists promoted protective effects (25). The development of oral TRPA1 antagonists, such as GDC-0334 (58), for clinical trials and evaluating their effects on human subjects with ischemia will provide insights into the function of this channel in various diseases associated with ischemia.

Like other reviews, this review article has limitations related to database searching. This includes missing relevant studies and the exclusion of non-English language studies. Because of the lack of clinical findings about TRPA1’s role in I/R, the present study focused on experimental research and the underlying molecular mechanisms. Moreover, most studies are about TRPA1’s role in cerebral, peripheral vascular, and myocardial infarctions. It is recommended to investigate other relevant topics including the role of TRPA1 on liver, eye, kidney, and other organs’ I/R injury for future studies.

## Conclusion

The present review article shows that TRPA1, similar to other TRP channels (59) may either have protective or deteriorative functions during ischemia. The controversies suggest that TRPA1 has a modulatory role in the body since its activation causes opposite reactions. In summary, TRPA1 is a promising target for ischemia. Its function might vary depending on the activation level, the ischemic region, and the extent and duration of ischemia. 

## Authors’ Contributions

A R Conceptualization and Writing – review & editing; MS A, A A and MS A Writing – original draft. All the authors have read and approved the final version of the manuscript.

## Conflicts of Interest

None.

## References

[1] Akbari G, Ali Mard S, Veisi A (2018). A comprehensive review on regulatory effects of crocin on ischemia/reperfusion injury in multiple organs. Biomed Pharmacother.

[2] Makhdoumi P, Roohbakhsh A, Karimi G (2016). MicroRNAs regulate mitochondrial apoptotic pathway in myocardial ischemia-reperfusion-injury. Biomed Pharmacother.

[3] Minutoli L, Puzzolo D, Rinaldi M, Irrera N, Marini H, Arcoraci V (2016). ROS-mediated NLRP3 inflammasome activation in brain, heart, kidney, and testis ischemia/reperfusion injury. Oxid Med Cell Longev.

[4] Kalogeris T, Baines CP, Krenz M, Korthuis RJ (2016). Ischemia/reperfusion. Compr Physiol.

[5] Maida CD, Norrito RL, Daidone M, Tuttolomondo A, Pinto A (2020). Neuroinflammatory mechanisms in ischemic stroke: Focus on cardioembolic stroke, background, and therapeutic approaches. Int J Mol Sci.

[6] Soler EP, Ruiz VC (2010). Epidemiology and risk factors of cerebral ischemia and ischemic heart diseases: similarities and differences. Curr Cardiol Rev.

[7] Vishwakarma VK, Upadhyay PK, Gupta JK, Yadav HN (2017). Pathophysiologic role of ischemia reperfusion injury: A review. J Indian Coll Cardiol.

[8] Alavi MS, Shamsizadeh A, Karimi G, Roohbakhsh A (2020). Transient receptor potential ankyrin 1 (TRPA1)-mediated toxicity: friend or foe?. Toxicol Mech Methods.

[9] Talavera K, Startek JB, Alvarez-Collazo J, Boonen B, Alpizar YA, Sanchez A (2020). Mammalian transient receptor potential TRPA1 channels: From structure to disease. Physiol Rev.

[10] Wu Y, Chen AW, Goodnough CL, Lu Y, Zhang Y, Gross ER (2021). IcyHot analgesic topical cream limits cardiac injury in rodents. Transl Res.

[11] Takahashi N, Kuwaki T, Kiyonaka S, Numata T, Kozai D, Mizuno Y (2011). TRPA1 underlies a sensing mechanism for O2. Nat Chem Biol.

[12] Etemad L, Karimi G, Alavi MS, Roohbakhsh A (2022). Pharmacological effects of cannabidiol by transient receptor potential channels. Life Sci.

[13] Takahashi N, Chen HY, Harris IS, Stover DG, Selfors LM, Bronson RT (2018). Cancer cells co-opt the neuronal redox-sensing channel TRPA1 to promote oxidative-stress tolerance. Cancer Cell.

[14] Paulsen CE, Armache JP, Gao Y, Cheng Y, Julius D (2015). Structure of the TRPA1 ion channel suggests regulatory mechanisms. Nature.

[15] Trevisani M, Siemens J, Materazzi S, Bautista DM, Nassini R, Campi B (2007). 4-Hydroxynonenal, an endogenous aldehyde, causes pain and neurogenic inflammation through activation of the irritant receptor TRPA1. Proc Natl Acad Sci U S A.

[16] Wang Z, Ye D, Ye J, Wang M, Liu J, Jiang H (2019). The TRPA1 channel in the cardiovascular system: Promising features and challenges. Front Pharmacol.

[17] Bugger H, Pfeil K (2020). Mitochondrial ROS in myocardial ischemia reperfusion and remodeling. Biochim Biophys Acta Mol Basis Dis.

[18] Wu L, Xiong X, Wu X, Ye Y, Jian Z, Zhi Z (2020). Targeting oxidative stress and inflammation to prevent ischemia-reperfusion injury. Front Mol Neurosci.

[19] Kim S, Hwang SW (2013). Emerging roles of TRPA1 in sensation of oxidative stress and its implications in defense and danger. Arch Pharm Res.

[20] Lu Y, Piplani H, McAllister SL, Hurt CM, Gross ER (2016). Transient receptor potential ankyrin 1 activation within the cardiac myocyte limits ischemia-reperfusion injury in rodents. Anesthesiology.

[21] Li S, Sun X, Wu H, Yu P, Wang X, Jiang Z (2019). TRPA1 promotes cardiac myofibroblast transdifferentiation after myocardial infarction injury via the calcineurin-NFAT-DYRK1A signaling pathway. Oxid Med Cell Longev.

[22] Li R, Liu R, Yan F, Zhuang X, Shi H, Gao X (2020). Inhibition of TRPA1 promotes cardiac repair in mice after myocardial infarction. J Cardiovasc Pharmacol.

[23] Lin L, Wang X, Yu Z (2016). Ischemia-reperfusion injury in the brain: mechanisms and potential therapeutic strategies. Biochem Pharmacol (Los Angel)..

[24] Jurcau A, Simion A (2021). Neuroinflammation in cerebral ischemia and ischemia/reperfusion injuries: from pathophysiology to therapeutic strategies. Int J Mol Sci.

[25] Pires PW, Earley S (2018). Neuroprotective effects of TRPA1 channels in the cerebral endothelium following ischemic stroke. Elife.

[26] Zaki OS, Nassar NN, Abdallah DM, Safar MM, Mohammed RA (2022). Cilostazol alleviates NLRP3 inflammasome-induced allodynia/hyperalgesia in murine cerebral cortex following transient ischemia: Focus on TRPA1/glutamate and Akt/dopamine/BDNF/Nrf2 trajectories. Mol Neurobiol.

[27] Hamilton NB, Kolodziejczyk K, Kougioumtzidou E, Attwell D (2016). Proton-gated Ca2+-permeable TRP channels damage myelin in conditions mimicking ischaemia. Nature.

[28] Giacco V, Flower G, Artamonova M, Hunter J, Padilla Requerey A, Hamilton NB (2023). Transient receptor potential Ankyrin-1 (TRPA1) agonists suppress myelination and induce demyelination in organotypic cortical slices. Glia.

[29] Lajoso W, Flower G, Giacco V, Kaul A, La Mache C, Brăban A (2021). Transient receptor potential ankyrin-1 (TRPA1) block protects against loss of white matter function during ischaemia in the mouse optic nerve. Pharmaceuticals (Basel).

[30] Zhou T, Li J, Cheng A, Zuo Z (2023). Desflurane post-treatment reduces hypoxic-ischemic brain injury via reducing transient receptor potential ankyrin 1 in neonatal rats. Neuroscience.

[31] Hoebart C, Kiss A, Pilz PM, Szabo PL, Podesser BK, Fischer MJM (2023). TRPA1 as target in myocardial infarction. Int J Mol Sci.

[32] Ahmadian Salami A, Alavi MS, Souri MS, Roohbakhsh A (2022). Involvement of the transient receptor potential A1 in morphine-induced conditioned place preference and physical dependence in mice. Can J Physiol Pharmacol.

[33] Andrei SR, Ghosh M, Sinharoy P, Damron DS (2019). Stimulation of TRPA1 attenuates ischemia-induced cardiomyocyte cell death through an eNOS-mediated mechanism. Channels (Austin).

[34] Ma Y, Iyer RP, Jung M, Czubryt MP, Lindsey ML (2017). Cardiac fibroblast activation post-myocardial infarction: current knowledge gaps. Trends Pharmacol Sci.

[35] Conklin DJ, Guo Y, Nystoriak MA, Jagatheesan G, Obal D, Kilfoil PJ (2019). TRPA1 channel contributes to myocardial ischemia-reperfusion injury. Am J Physiol Heart Circ Physiol.

[36] Üstünel L, Özgüler IM (2021). The effects of iloprost and beta3 receptor agonist on TRPA1 and TRPC1 immunreactivity in an experimental lower extremty ischemia-reperfusion injury model. Turk J Med Sci.

[37] Ma S, Wang DH (2021). Knockout of Trpa1 exacerbates renal ischemia-reperfusion injury with classical activation of macrophages. Am J Hypertens.

[38] So K, Tei Y, Zhao M, Miyake T, Hiyama H, Shirakawa H (2016). Hypoxia-induced sensitisation of TRPA1 in painful dysesthesia evoked by transient hindlimb ischemia/reperfusion in mice. Sci Rep.

[39] Klafke JZ, da Silva MA, Rossato MF, de Prá SD, Rigo FK, Walker CI (2016). Acute and chronic nociceptive phases observed in a rat hind paw ischemia/reperfusion model depend on different mechanisms. Pflugers Arch.

[40] De Logu F, De Prá SD, de David Antoniazzi CT, Kudsi SQ, Ferro PR, Landini L (2020). Macrophages and Schwann cell TRPA1 mediate chronic allodynia in a mouse model of complex regional pain syndrome type I. Brain Behav Immun.

[41] Baek S-S, Kim S-H (2016). Treadmill exercise ameliorates symptoms of Alzheimer disease through suppressing microglial activation-induced apoptosis in rats. J Exerc Rehabil.

[42] Sasaki A, Mizoguchi S, Kagaya K, Shiro M, Sakai A, Andoh T (2014). A mouse model of peripheral postischemic dysesthesia: involvement of reperfusion-induced oxidative stress and TRPA1 channel. J Pharmacol Exp Ther.

[43] Hiyama H, Yano Y, So K, Imai S, Nagayasu K, Shirakawa H (2018). TRPA1 sensitization during diabetic vascular impairment contributes to cold hypersensitivity in a mouse model of painful diabetic peripheral neuropathy. Mol Pain.

[44] Xing J, Lu J, Li J (2015). TRPA1 mediates amplified sympathetic responsiveness to activation of metabolically sensitive muscle afferents in rats with femoral artery occlusion. Front Physiol.

[45] Xing J, Li J (2017). TRPA1 function in skeletal muscle sensory neurons following femoral artery occlusion. Cell Physiol Biochem.

[46] Xing J, Li J (2017). Proteinase-activated receptor-2 sensitivity of amplified TRPA1 activity in skeletal muscle afferent nerves and exercise pressor reflex in rats with femoral artery occlusion. Cell Physiol Biochem.

[47] Pizzimenti S, Ciamporcero ES, Daga M, Pettazzoni P, Arcaro A, Cetrangolo G (2013). Interaction of aldehydes derived from lipid peroxidation and membrane proteins. Front Physiol.

[48] Lin Y, Chen Z, Tang J, Cao P, Shi R (2018). Acrolein contributes to the neuropathic pain and neuron damage after ischemic-reperfusion spinal cord injury. Neuroscience.

[49] Qin Q, Yu N, Gu Y, Ke W, Zhang Q, Liu X (2022). Inhibiting multiple forms of cell death optimizes ganglion cells survival after retinal ischemia reperfusion injury. Cell Death & Disease.

[50] Souza Monteiro de Araújo D, De Logu F, Adembri C, Rizzo S, Janal MN, Landini L (2020). TRPA1 mediates damage of the retina induced by ischemia and reperfusion in mice. Cell Death Dis.

[51] Araújo DSM, Miya-Coreixas VS, Pandolfo P, Calaza KC (2017). Cannabinoid receptors and TRPA1 on neuroprotection in a model of retinal ischemia. Exp Eye Res.

[52] Malek M, Nematbakhsh M (2015). Renal ischemia/reperfusion injury; from pathophysiology to treatment. J Renal Inj Prev.

[53] Wu CK, Wu CL, Lee TS, Kou YR, Tarng DC (2021). Renal tubular epithelial TRPA1 acts as an oxidative stress sensor to mediate ischemia-reperfusion-induced kidney injury through MAPKs/NF-κB signaling. Int J Mol Sci.

[54] Den Hengst WA, Gielis JF, Lin JY, Van Schil PE, De Windt LJ, Moens AL (2010). Lung ischemia-reperfusion injury: a molecular and clinical view on a complex pathophysiological process. Am J Physiol Heart Circ Physiol.

[55] Taylor-Clark TE, Undem BJ (2011). Sensing pulmonary oxidative stress by lung vagal afferents. Respir Physiol Neurobiol.

[56] Gu X, Yu N, Pang X, Zhang W, Zhang J, Zhang Y (2019). EXPRESS: Products of oxidative stress and TRPA1 expression in the brainstem of rats after lung ischemia-reperfusion injury. Pulm Circ.

[57] Zhang XH, Qi HX, Xu DS, Pang XC, Wang CY, Yu WJ (2019). Expression of proteinase-activated receptor-2 and transient receptor potential A1 in vagal afferent nerve of rat after lung schemia-reperfusion injury. J Biol FRegul Homeost Agents.

[58] Balestrini A, Joseph V, Dourado M, Reese RM, Shields SD, Rougé L (2021). A TRPA1 inhibitor suppresses neurogenic inflammation and airway contraction for asthma treatment. J Exp Med.

[59] Hakimizadeh E, Shamsizadeh A, Roohbakhsh A, Arababadi MK, Hajizadeh MR, Shariati M (2017). TRPV1 receptor-mediated expression of Toll-like receptors 2 and 4 following permanent middle cerebral artery occlusion in rats. Iran J Basic Med Sci.

